# Seeing the Supracolloidal Assemblies in 3D: Unraveling High-Resolution Structures Using Electron Tomography

**DOI:** 10.1021/acsmaterialsau.3c00067

**Published:** 2023-11-09

**Authors:** Nonappa

**Affiliations:** Faculty of Engineering and Natural Sciences, Tampere University, FI-33720 Tampere, Finland

**Keywords:** Electron tomography, nanoparticles, self-assemblies, biohybrids, colloids, superlattices

## Abstract

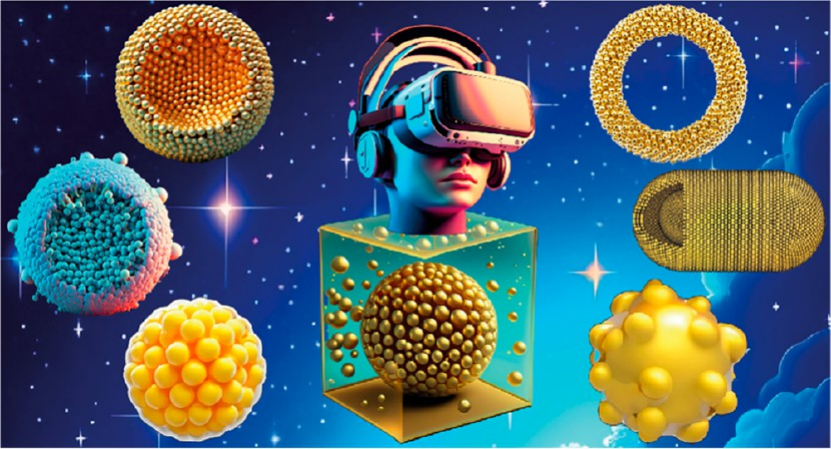

Transmission electron microscopy (TEM) imaging has revolutionized modern materials science, nanotechnology, and structural biology. Its ability to provide information about materials’ structure, composition, and properties at atomic-level resolution has enabled groundbreaking discoveries and the development of innovative materials with precision and accuracy. Electron tomography, single particle reconstruction, and microcrystal electron diffraction techniques have paved the way for the three-dimensional (3D) reconstruction of biological samples, synthetic materials, and hybrid nanostructures at near atomic-level resolution. TEM tomography using a series of two-dimensional (2D) projections has been used extensively in biological science, but in recent years it has become an important method in synthetic nanomaterials and soft matter research. TEM tomography offers unprecedented morphological details of 3D objects, internal structures, packing patterns, growth mechanisms, and self-assembly pathways of self-assembled colloidal systems. It complements other analytical tools, including small-angle X-ray scattering, and provides valuable data for computational simulations for predictive design and reverse engineering of nanomaterials with the desired structure and properties. In this perspective, I will discuss the importance of TEM tomography in the structural understanding and engineering of self-assembled nanostructures with specific emphasis on colloidal capsids, composite cages, biohybrid superlattices with complex geometries, polymer assemblies, and self-assembled protein-based superstructures.

## 1 Introduction

Transmission electron microscopy (TEM) is an indispensable tool for studying the structure and properties of materials at the atomic level. Since its invention in 1931,^1,2^ TEM has developed rapidly, with advances in instrumentation,^3^ electron sources,^4,5^ detectors,^6^ specimen preparation,^7^ imaging, and imaging processing methods.^6^ These advances have enabled TEM to provide unprecedented details of materials, including their morphology, internal structures, elemental composition, spectroscopic properties, and mechanical behaviors.^8−11^ TEM has continuously evolved from a tool to study ultrasmall objects’ morphology, size, and shape to complete structure determination of biological and synthetic materials at near-atomic resolution.^12−14^ Historically, TEM imaging has been used extensively in biology to study the structural details of viruses and bacteria.^15−19^ Furthermore, nanoparticle-enabled immunolabeling has been used for studying the interactions between different biomolecules and identifying the location of specific biomarkers within cells.^20−22^ Early attempts to develop biological specimen preparation methods have used heavy metal atoms for metal shadowing and negative staining.^23^ Furthermore, methods including chemical fixation,^24^ critical point drying,^25^ and sugar (glucose and trehalose) embedding,^26,27^ have provided minimum drying artifacts resulting in high-resolution imaging of two-dimensional (2D) crystals of biomolecules, including most challenging membrane proteins.^28^ The development of cryogenic TEM (cryo-TEM) specimen preparation, single particle reconstruction (SPR) methods, and microcrystal electron diffraction (microED) has transformed TEM-based bioimaging.^29−37^ Today, cryo-TEM allows the preparation of a broad range of biological specimens and synthetic soft materials. More recently, the application of cryo-TEM has expanded to study battery materials, making TEM one of the most valuable imaging and analytical tools.^38^

In materials science, TEM imaging has played a significant role in understanding metal nanoparticle (MNP) size, shape, and properties. In the 1950s, Turkevich et al. extensively studied the gold nanoparticle (AuNP) size, shape, nucleation, and growth in colloidal gold sols using TEM imaging.^39^ Today, advanced aberration-corrected TEM and scanning transmission electron microscopy (STEM) imaging offer unprecedented details on metal nanoparticle size, shape, atom dislocation, grain boundaries, spectroscopy, elemental mapping, and mechanical properties.^40−45^ More importantly, recent reports have demonstrated the 3D structure determination of atomically precise gold nanoparticles using single particle reconstruction,^46^ and microED.^47^

TEM and other analytical tools, synthetic methodologies, and computational simulations have contributed to the development of MNPs with a high degree of control over size, shape, and composition. Therefore, NPs have emerged as excellent colloidal-level building blocks for self-assembly.^48^ Specifically, MNPs capped with organic ligands offer enhanced stability, solubility, and directional interactions.^49^ The self-assembly of MNPs into various superstructures with complex shapes and geometries, including capsids, supraparticles, colloidal cages, and microwires, has been reported.^50−54^ The self-assembled structures offer enhanced optical properties, catalytic efficiency, mechanical strength, stability, nanoconfinement for selective encapsulation of biologically active materials, act as nanoflasks for stereospecific organic reactions, and display amplified luminescence.^55,56^ Therefore, understanding their morphology, internal structures, and packing patterns is critical for navigating the structure–property relationship. Furthermore, a detailed understanding of the 3D structures is crucial for the engineering and predictive design of new colloidal structures with the desired properties. However, conventional TEM imaging produces 2D projections of 3D objects. The 2D projections provide extremely valuable information about the specimen and contain high-resolution structural details. However, due to intrinsic limitations in the thickness of the specimen and the superimposed nature of the images, limited information can be gained solely using 2D projections of self-assembled superstructures. Furthermore, the 2D projections depend on the object’s orientation under investigation. Thus, retrieving the details in the third dimension is crucial to gaining structural insights. Stereo images, i.e., collecting images with two slightly different viewing angles are simple forms to visualize 3D structures. However, stereo images cannot convey the complete internal structural details. In this context, TEM tomography has evolved as a revolutionary technique to study and determine 3D structures of a wide range of synthetic, biological, and hybrid materials.

The term tomography is derived from “tomos”, meaning “slices or sections”, and “graphe”, meaning “drawing” or, in other words, imaging by sections. Tomography has a history of more than a century. In 1917, Radon first proposed the mathematical formulation for reconstructing 3D volume or density maps using a set of 2D projections.^57^ However, the application of tomography emerged only in the 1950s, particularly in astronomy.^58,59^ Tomography rapidly gained momentum in medical imaging, and the early work of Cormack and Hounsfield led to X-ray computed tomography (CT).^60,61^ The concept of tomography was introduced to TEM imaging by de Rosier and Klug in 1968, who reported the 3D density map of the tail of a T4 bacteriophage using a set of TEM images.^62^ In the same year, Hart reported the polytropic montage of tobacco mosaic virus (TMV) particles and showed that the unstained sample could provide high-resolution details compared to single projections.^63^ TMV, T4 bacteriophage, and ferritin particles were studied between the 1960s and 1980s using electron microscopy and protein crystallography.^64−66^ The introduction of STEM tomography has further allowed the high-resolution 3D imaging and quantitative analysis of materials under extreme conditions.^67−69^

**Figure 1:**
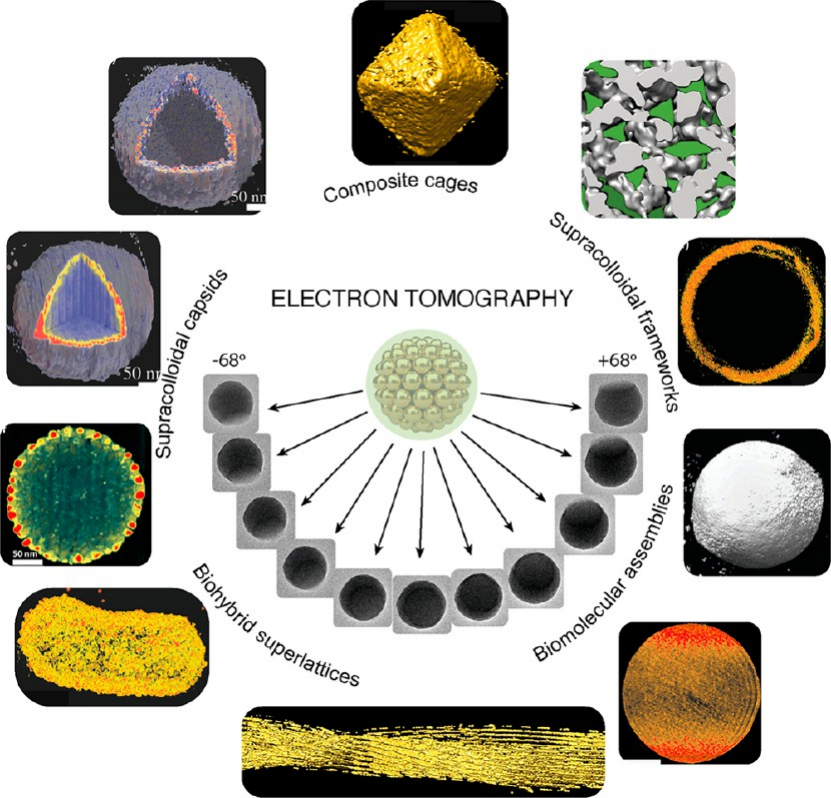
Electron tomography of self-assembled superstructures discussed in this Perspective.

TEM tomography relies on a series of 2D projections (i.e., tilt series) collected across different viewing angles by tilting the specimen holder with a known increment angle (Figure 1). The 2D projections are computationally aligned using cross-correlation methods or preloaded fiducial markers. Several methods have been used to collect tilt series, including random conical tilt, increment angle, increment slope, and dual-axis tilt. More importantly, atomic resolution tomography, fast STEM, and EDS tomography methods have recently been implemented to study 3D structures of individual MNPs, atom dislocation, elemental composition, twinning, and thermal phase transition of nanoparticles.^70−78^ Furthermore, highly sophisticated 4D electron microscopy imaging has been demonstrated.^79−81^ Extensive discussion on the application of various TEM and STEM tomography methods is beyond the scope of this article, and excellent reviews on fundamental concepts of electron tomography, theoretical insights, and examples can be found in several recent reviews.^8,9,67,75^ In this Perspective, I will discuss the application of TEM tomography in unraveling the high-resolution 3D details of self-assembled soft colloidal superstructures (Figure 1). I will focus on four key areas of applications: (i) understanding the morphology and internal structures of supracolloidal assemblies, (ii) gaining insights on self-assembly mechanistic details of NP frameworks to understand structure–property relationships (e.g., enhanced optical and catalytic properties), (iii) providing self-assembly mechanism, growth patterns and unit cell parameters of biohybrid superlattices and composite frameworks, and (iv) experimental methods to study soft biomolecular assemblies. I will show some of the selected examples of supracolloidal spherical and rod-like capsids, NP frameworks, nanocluster (NC) frameworks, NP-NC composites, toroidal structures, biohybrid superlattices, and soft biomolecular assemblies (Figure 1).

## 2 Supracolloidal Capsids

**Figure 2:**
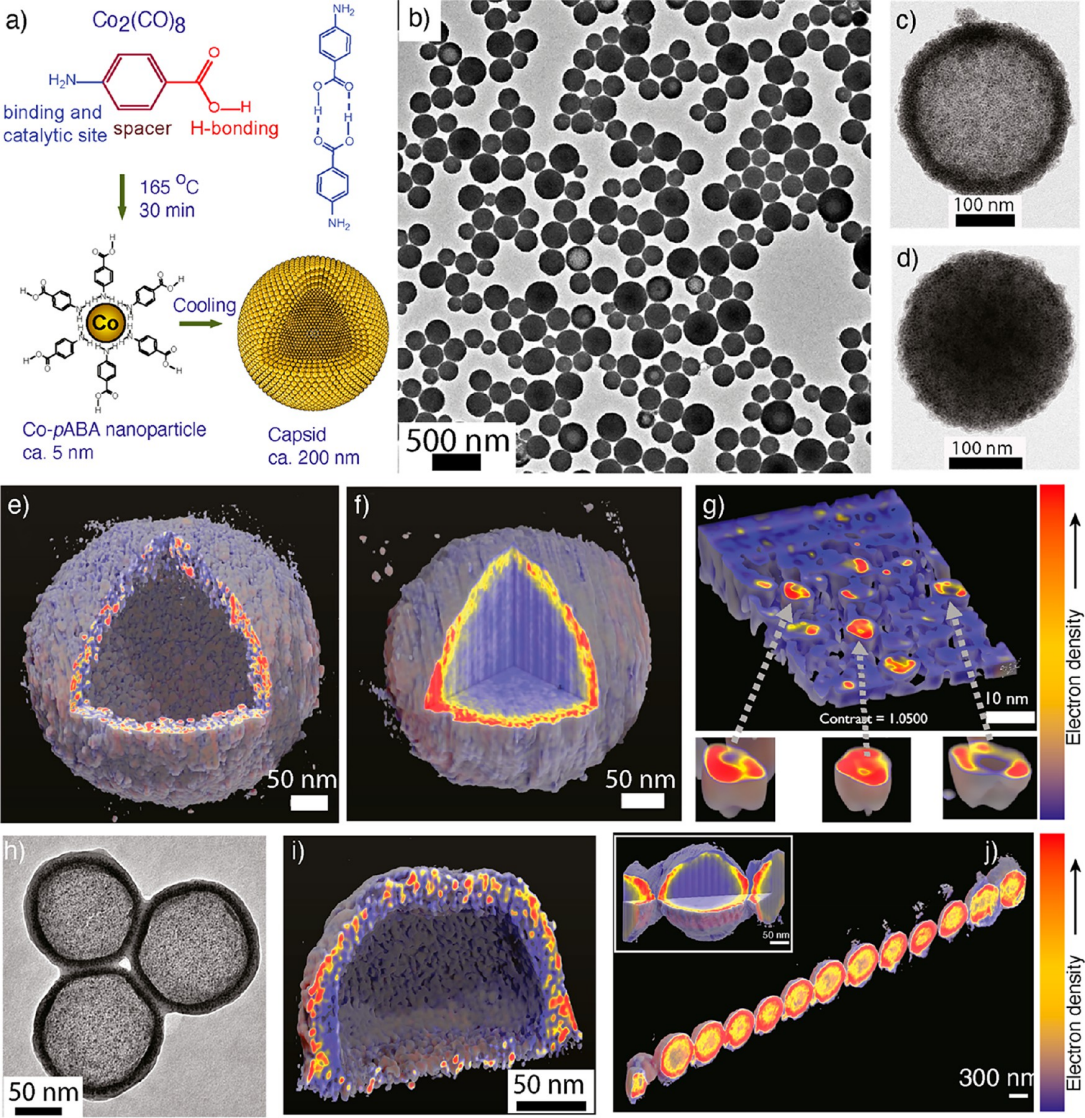
Supracolloidal capsids. (a) Chemical structures, synthesis, and *in situ* self-assembly of CoNPs. (b) TEM image of the as-synthesized CoNP capsids in 1,2-DCB. (c,d) Higher magnification images of individual capsids showing core–shell structures. (e) 3D reconstructed image of a capsid with an empty interior and ∼20 nm multilayered shell. (f) 3D reconstructed image of a capsid containing amorphous materials in the core and ∼20 nm multilayered shell. (g) 3D reconstructed cross-sectional view of the shell showing individual CoNPs and the voids filled with amorphous materials. (h) TEM images of capsids after solvent exchange to acetone showing core–shell structure. (i) 3D reconstructed image of acetone-treated capsids showing an empty core and a deformed shell. (j) 3D reconstructed capsid chains formed under the magnetic field suggest capsids’ structure remains intact (inset shows the 3D reconstruction of a single capsid from the chain at higher magnification). Reproduced with permission from ref (85). Copyright 2017 John Wiley & Sons.

In nature, virus capsids represent a fascinating example of genetic economy, efficiency, and error-free structure formation.^82^ They display subunit-based self-assembly and are inspirations for synthetic self-assembled systems. Furthermore, capsids undergo facile reversible assembly disassembly by tuning the chemical or other environmental conditions.^83^ Their reversible nature also offers routes for selective and size-dependent encapsulation of various materials, including nanoparticles.^84^ However, biological particles are delicate and operate only under narrow experimental conditions. Mimicking capsid-like assemblies using metal nanoparticles offers structure formation under a broad range of experimental conditions for materials with unique chemical, optical, and magnetic properties. Nonappa et al. reported *in situ*, template-free, and reversible self-assembly of superparamagnetic cobalt nanoparticles (CoNPs) into spherical capsids (Figure 2).^85^ The capsids were prepared using the heating-up synthesis of a mixture of dicobalt octacarbonyl Co_2_(CO)_8_ and *p*-aminobenzoic acid (*p*ABA) in 1,2-dichlorobenzene (1,2-DCB) solvent (Figure 2a). The TEM imaging of the specimen prepared from the reaction mixture showed capsids with an average diameter of 200 nm. TEM images suggested two morphologies (Figure 2b): (i) capsids with a contrast difference between the core and the shell (Figure 2c) and (ii) capsids with a core and shell with similar contrast (Figure 2d). Furthermore, cryo-TEM imaging and dynamic light scattering (DLS) analysis confirmed the presence of stable capsids in the solution. The capsids were readily disassembled into individual CoNPs (*d* ∼ 4–10 nm) when treated with methanol. Importantly, reassembled spherical capsids were obtained when the methanol-treated individual CoNPs were redispersed in 1,2-DCB. Reversible assembly disassembly was also observed when the sample was subjected to a heating–cooling cycle. Interestingly, upon exchanging the solvent from 1,2-DCB to acetone, all particles turned into capsids with flexible shells with a clear difference in the contrast between the core and shell (Figure 2h,i).

Solvent exchange studies, spectroscopic analysis, and computational simulations suggested that the self-assembly is driven by the hydrogen-bonding dimerization of carboxylic acid groups of *p*ABA ligands. Surprisingly, the magnetic measurement of capsids revealed superparamagnetic properties with a magnetic diameter of ∼3.2 nm, which corresponds to the magnetic core of individual NPs (neglecting the nonmagnetic oxide layer). The results suggest that the capsids are self-assembled superstructures, not random aggregates. Furthermore, the intrinsic superparamagnetic property of individual NPs is retained in the capsids. More importantly, even a low magnetic field of 0.65T (neodymium magnet) induced one-dimensional chains or necklace-like assemblies of capsids (Figure 2j). The capsid chains remained stable once the magnetic field was removed and were resistant to mechanical perturbation. This is attributed to magnetic dipole-induced attraction and intercapsid hydrogen bonding. Importantly, in capsids, individual CoNPs are magnetically noninteracting and purely interacting via hydrogen bonding of surface ligands.

Electron tomography of as-synthesized capsids in 1,2-DCB, acetone-treated capsids, and magnetic field-treated capsid chains revealed some key insights into morphology, internal structures, and packing patterns. Importantly, these observations provide complementary evidence of self-assembly and the rationale behind their morphological differences. The 3D reconstruction revealed that the capsids have ∼20 nm multilayered shells. However, the capsids with low-contrast cores revealed empty interiors (Figure 2e). On the other hand, the capsids with uniform core–shell contrast displayed an interior filled with amorphous material (Figure 2f). The solvent exchange and nuclear magnetic resonance (NMR) spectroscopy analyses suggest that the core was filled with excess and unreacted *p*ABA ligands. Notably, the multilayered shell had no regular packing patterns. A subtomography analysis of the shell suggests that the excess unreacted ligands are distributed or trapped between the nanoparticle cavities (Figure 2g). This was also supported using density functional theory (DFT) calculation studies of a model Co-*p*ABA cluster. 3D reconstruction of acetone-treated particles showed a similar shell thickness but with a hollow core. Furthermore, the shell was porous and deformed. This suggests that excess ligands trapped in the capsid core and the shell were removed when treated with acetone (Figure 2h,i).

**Figure 3:**
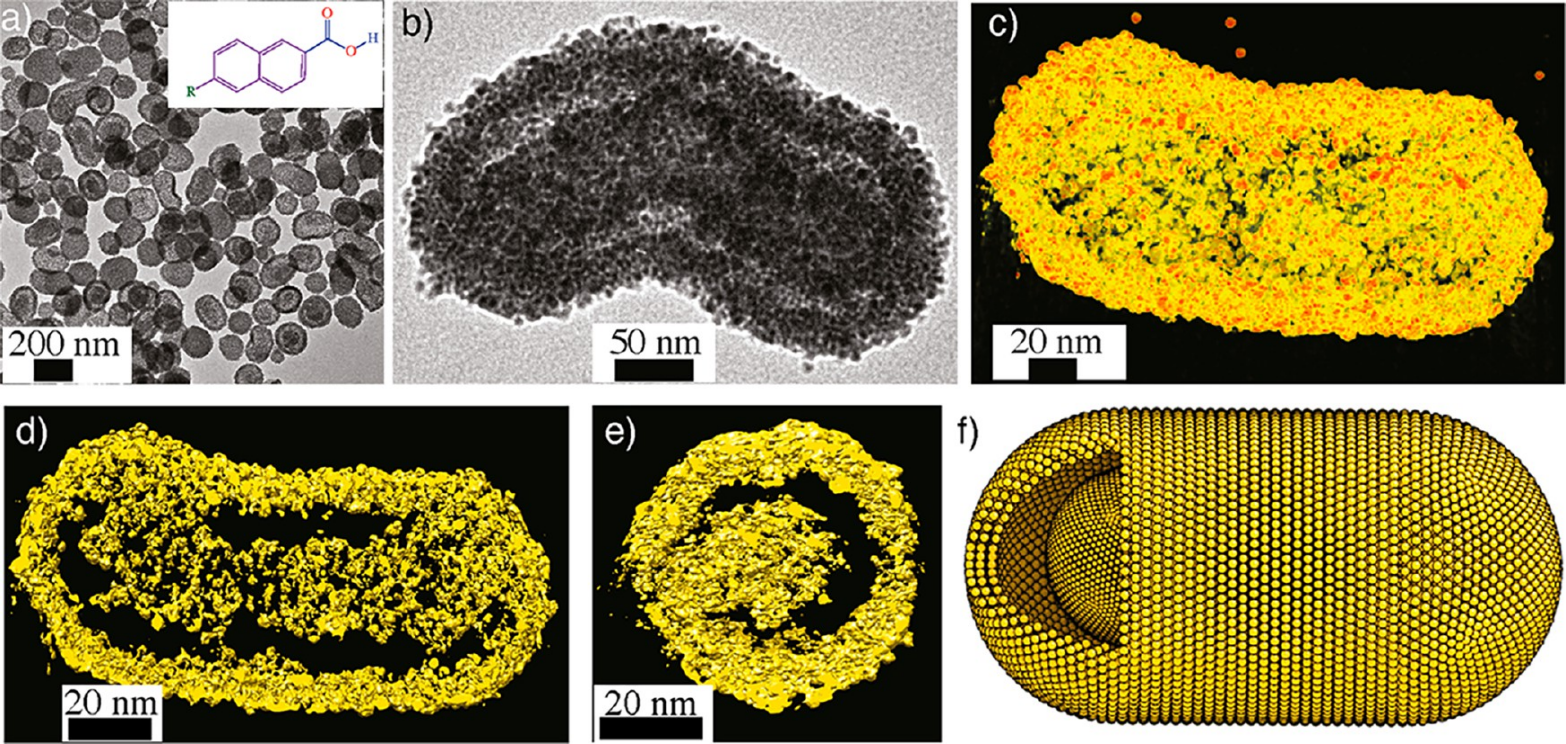
Rod-like capsids. (a) TEM image of CoNP capsids prepared using *p*ANA ligands (inset shows the chemical structure of the *p*ANA ligand, R = NH_2_). (b) Higher magnification TEM image of a capsid showing core–shell structure. (c) 3D reconstructed structure of the capsid. (d,e) Cross-sectional views showing rod-in-rod architecture. (f) Schematic representation of a rod-in-rod capsid. Reproduced with permission from ref (86). Copyright 2018 John Wiley & Sons.

In another study, capsids were prepared using Co_2_(CO)_8_ and 6-amino-2-naphthoic acid (*p*ANA) in 1,2-DCB. Unlike *p*ABA-mediated CoNP capsid formation, *p*ANA-capped CoNPs resulted in unique rod-like capsids with an average length of 200 nm and a lateral diameter of 100 nm (Figure 3a).^86^

The rod-like capsids are composed of ∼20 nm shells. Furthermore, the core of the capsids contains a rod-like nanoparticle assembly (*d* ≈ 50 nm), i.e., rod-in-rod morphology (Figure 3b). The 3D reconstruction revealed that the capsids comprise shells consisting of a few layers of nanoparticles with a shell thickness of 20 nm (Figure 3c). The sizes of the individual building blocks were similar to those of the Co-*p*ABA NPs and were superparamagnetic. Furthermore, the rod-like assembly within the interior of the capsid is composed of individual CoNPs similar to the shell (Figure 3d). The interspatial distance between the shell and the nanorod was ∼20–25 nm (Figure 3e,f). This suggests that by careful ligand engineering, the structure of the capsids can be tuned toward novel assemblies. While the formation of rod-like structures is interesting, what drives such structures’ growth is unclear. The formation of rod-shaped structures requires symmetry breaking, which can arise from the specific properties of the ligands, their interaction, and stacking. A more detailed study is needed to understand the phenomenon of this anisotropic growth. Time-resolved TEM, *in situ* liquid cell TEM, and tomography data-assisted computational simulations may shed more insight into the structure formation mechanism and predictive design of novel colloidal capsids.

**Figure 4:**
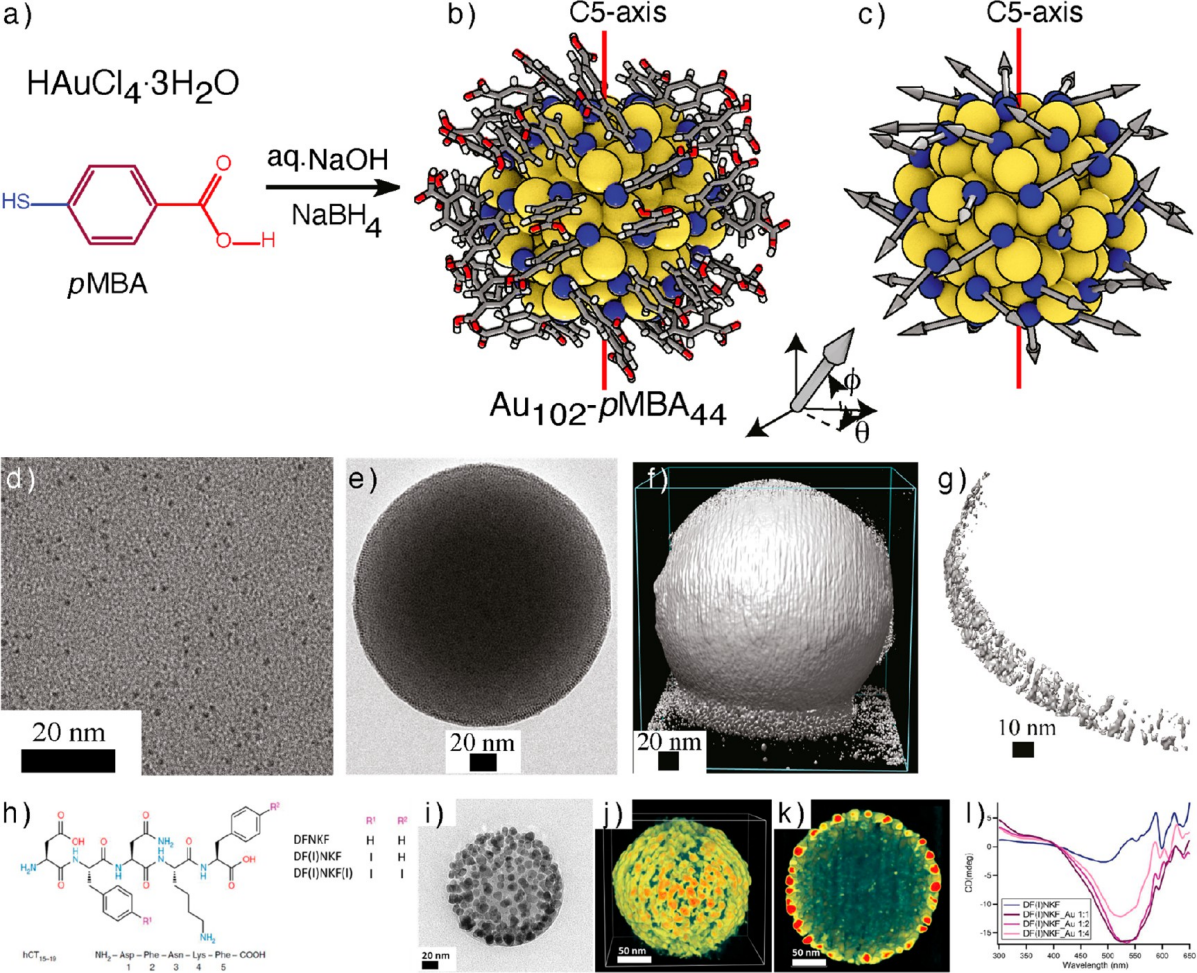
Self-assembled capsids with monolayer shells. (a) Synthesis of the Au_102_-*p*MBA_44_ NC. (b) X-rays single crystal structure of Au_102_-*p*MBA_44_ NC (yellow: Au, blue: S, red: O, gray: C, and white: H). (c) Ligands are represented in arrows to determine their location and orientation in a 3D coordinate system. (d) TEM image of Au_102_-*p*MBA_44_ NCs. (e) TEM image of the self-assembled colloidal capsid. (f) 3D reconstructed structure of the capsid. (g) Part of the shell showing monolayer thickness. Panels a-g reproduced with permission from ref.^88^ Copyright 2016 John Wiley & Sons. (h) Chemical structures of DFNKF peptides. (i) TEM image of an *in situ* generated gold-peptide capsid. (j) 3D reconstructed structure of capsid. (k) cross-sectional view showing monolayer shell with amorphous peptides in the interior. (l) CD spectrum of DF(I)NKF peptides when treated with different ratios of Au. Panels (h)–(l) reproduced with permission under a Creative Commons license (CC-BY 4.0) from ref (89). Copyright 2019 American Chemical Society.

The above results showed well-defined capsid formation using nonuniform NP building blocks via hydrogen bonding interaction. The self-assembly of CoNPs was rapid as the synthesis was performed at a high temperature (165 °C) in a nonpolar solvent. In nonpolar solvents, the carboxylic acid remains as a monomer at high temperatures, and as the temperature is lowered, it undergoes rapid dimerization. This might lead to less-ordered multilayer assemblies with excess ligands trapped inside and between the voids of the shells. Identifying uniform building blocks, controllable self-assembly conditions, and tunable inter-NP interactions is crucial to understanding capsid formation. In this context, atomically precise monolayer thiol-protected noble metal nanoclusters (NCs) have emerged as attractive building blocks for self-assembly.^86,87^ Because of their exactly defined number of metal atoms and ligands, they offer controlled self-assembly. Nonappa et al. reported the template-free self-assembly of *p*-mercaptobenzoic acid (*p*MBA) capped atomically precise gold nanoclusters (AuNCs), Au_102_-*p*MBA_44_ into 2D colloidal crystals and supracolloidal capsids (Figure 4).^88^ Au_102_-*p*MBA_44_ NC contains 102 gold atoms and 44 *p*MBA ligands (Figure 4a–c). When the carboxylic acid groups of all ligands are protonated, the NCs are dispersible in methanol and insoluble in water. However, partial deprotonation of carboxylic acid groups (∼22) imparts water solubility with an excellent colloidal stability. Therefore, selecting the proper self-assembly conditions can enable a delicate balance between attractive hydrogen bonding (carboxylic acid dimerization) and electrostatic repulsion (negatively charged carboxylates). Furthermore, the spherical coordinate system indicated that in Au_102_-*p*MBA_44,_ ligands are anisotropically distributed with a preferential orientation toward the equatorial plane of the NC. Notably, the deprotonation of Au_102_-*p*MBA_44_ leads to patchy negative charges imparting amphiphilic properties to the NCs. Therefore, the patchy and anisotropic distribution allows symmetry breaking, resulting in lower-dimensional structures such as 2D colloidal crystals. By creation of defects, curvature can be induced to obtain spherical structures.

When the aqueous dispersion of the partially deprotonated Au_102_-*p*MBA_44_ NC was sequentially dialyzed against methanol, it resulted in 2D colloidal crystals. However, spherical capsids were formed when an aqueous dispersion of NC was rapidly added to methanol (Figure 4e). Tomographic reconstruction suggested that that shell was monolayer thick. i.e., one nanoparticle (∼2.69 nm) thick (Figure 4f,g). While there is little evidence of what stabilizes the interior of such structures, it is likely the solvent or excess organic residue in the interior. The NC also forms ellipsoidal capsids with monolayer shells. The next question is whether the capsids with monolayer shells can be obtained using nonuniform NP building blocks.

Pigliacelli et al. utilized iodinated amyloidogenic peptides for *in situ* AuNP synthesis and templated assembly of chiroptically active capsid-like structures.^89^ The modified human calcitonin derived DFNKF peptide fragments were used as ligands and templates. The *para* position of either one or both phenylalanine (F) residues of the DNFKF peptides was substituted with iodine (Figure 4h). Iodination promotes the self-assembly of the peptides and simultaneously acts as a template for the deposition of Au(III) ions. This approach allows Au-mediated C–I activation to promote spontaneous nanoparticle formation on the surface of the templated superstructure. Spherical particles were produced when Au(III) salts were mixed with iodinated DNFKF peptides in aqueous media. The core was composed of peptide, and the surface was covered with Au ions, supported by STEM EDS spectra. Upon heating the aqueous mixture for 60–180 min, surface plasmon resonance peaks around 562 nm were observed, suggesting *in situ* nanoparticle formation. TEM image of the resulting structure displayed spherical superstructures (50–200 nm) composed of 6–10 nm AuNPs (Figure 4i). Electron tomography of the resulting superstructure revealed a spherical capsid-like structure (Figure 4j). The spherical particles displayed a monolayer shell of nanoparticles placed with uniform internanoparticle distance (Figure 4k). The core contained an amorphous, less dense interior. These results suggest that capsids with monolayer shells can be achieved via a templated approach using nonuniform building blocks.

By comparing the 3D reconstructions of the above four examples of self-assembled NP-based capsids, one can conclude the following. First, the nonuniform building blocks with directional hydrogen bonding ligands allow spherical template-free capsids. However, the capsids are multilayered without any regular packing patterns of NPs. Second, atomically, precise NCs containing hydrogen bonding ligands result in capsids with a well-defined monolayer shell. The shell has highly ordered packing patterns of the individual NCs. Finally, using nonuniform NP building blocks, capsids with monolayer shells can be achieved under a templated approach. Therefore, the size uniformity of the NPs and self-assembly conditions affect the resulting superstructures. TEM tomography provides high-resolution details on morphology, internal structures, and packing patterns in nonuniform and noncrystalline colloidal structures.

## 3 Nanoparticle Frameworks

**Figure 5:**
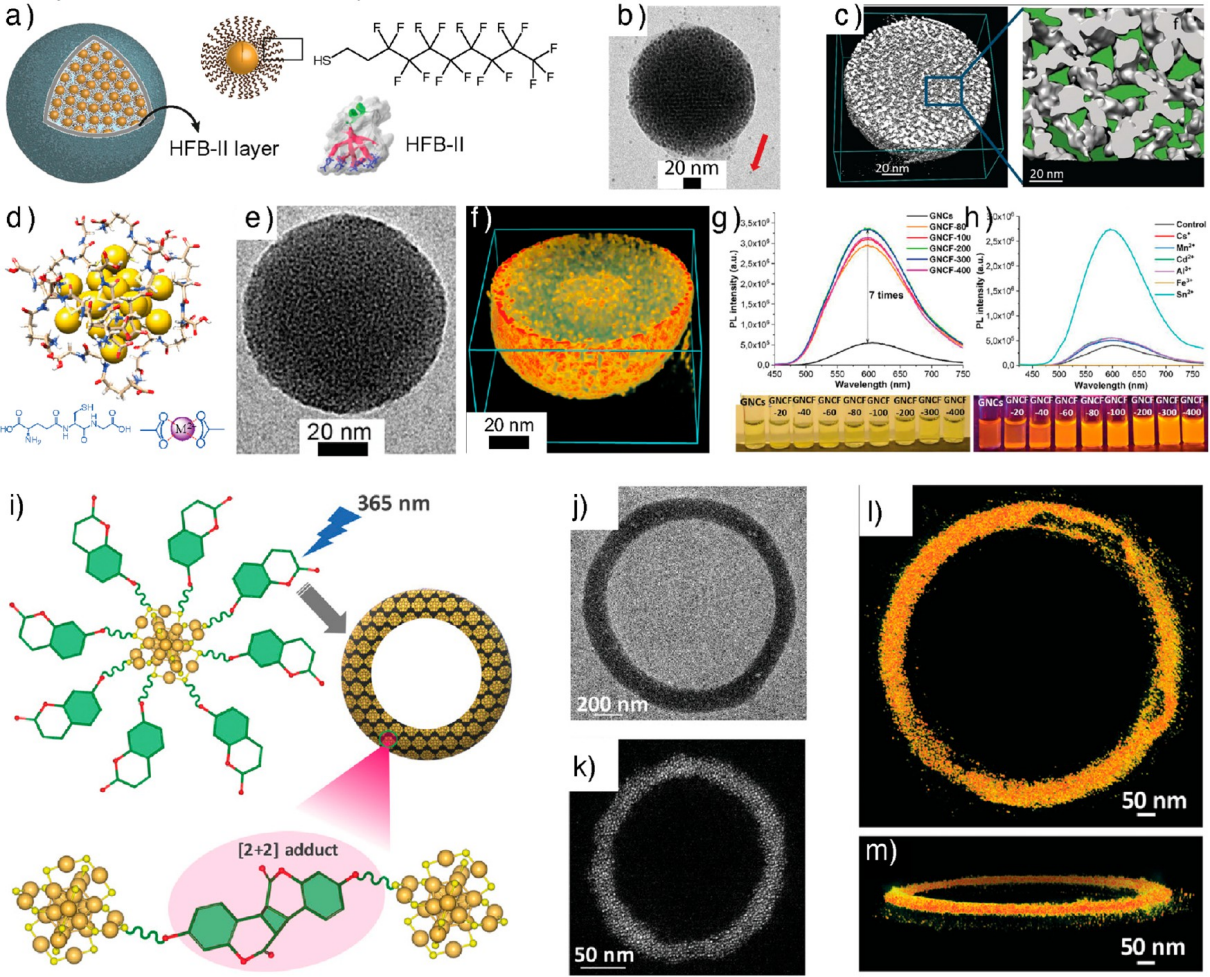
Nanoparticle frameworks. (a) Schematic representation of HFB-II mediated assembly of fluorinated AuNPs into supraparticles. (b) TEM image of a supraparticle. (c) Cross-sectional view of a 3D reconstructed FSP and a magnified view showing inter-NP voids for selective encapsulation. Panels (a)–(c) reproduced with permission from ref (90). Copyright 2017 John Wiley & Sons. (d) Structure of GSH-capped AuNC. (e) TEM image of an NC framework. (f) 3D reconstructed structure showing densely packed NCs. (g) Shows the change in PL intensity as a function of metal ion concentration. (h) shows the change in PL intensity when different divalent metal ions were added. Panels (d)–(h) reproduced with permission from ref (92). Copyright 2019 John Wiley & Sons. (i) Chemical structures and schematic representation of dynamic covalent chemistry induced toroid formation of coumarin thiol capped AuNCs. (j) TEM image of a toroid. (k) DF-STEM image of a toroid. (l,m) 3D reconstructed image of a toroid viewed at different orientations. Panels i-m reproduced with permission from ref (94). Copyright 2023 John Wiley & Sons.

The self-assembled capsids provide inspiration to investigate whether adding additional interactions or components can achieve even more ordered and compact arrangements of NPs instead of core–shell structures. Such self-assembled NP-based superstructures allow inter-NP compartmentalization. The compartmentalization offers nanoconfinement for selective encapsulation of small molecules, controlled drug delivery, and voids for chemical reactions by acting as nanoflasks.^48−56^ Pigliacelli et al. reported the self-assembled fluorous supraparticles (FSPs) to efficiently encapsulate poorly water-soluble fluorinated drugs (Figure 5a).^90^

The FSPs were fabricated using AuNPs capped with 1*H*,1*H*,2*H*,2*H*-perfluorodecanethiol (PFDT) ligands in the presence of the film-forming protein hydrophobin-II (HFB-II). Two types of NPs, viz., spherical AuNCs with an average diameter of 1.6 ± 0.6 nm and plasmonic AuNPs with an average diameter of 3.8 ± 0.8 nm, were used to study the effect of size on compartmentalization. Cryo-TEM imaging suggested the spherical nature of the SPs with diameters in the range 30–80 nm (Figure 5b). The SPs were observed for both NCs and NPs. The SPs comprised a NP core and multilayered protein (HFB-II) shell with an average 5–10 nm thickness. The SAXS spectrum of a water dispersion of SPs obtained from AuNCs showed two structure peaks at 2.1 and 3.9 nm^–1^, with an interparticle distance of about 3 nm. On the other hand, the SAXS spectrum of SPs obtained from AuNPs showed a less ordered structure with a SAXS pattern characterized by only one Bragg peak, corresponding to an average interparticle distance of the confined NPs of about 5.2 nm. Tomographic reconstruction of both SPs revealed a spherical morphology with a densely packed array of individual building blocks (Figure 5c). For SPs obtained from AuNPs, a densely packed array of NPs with intricate voids was formed. AuNC-containing SPs also showed a similar organization. Even though identical ligands were used in AuNCs and AuNPs, their core size differences resulted in different void spaces. AuNCs led to a more efficient and ordered packing with smaller voids, which agrees with the SAXS analysis results. The results also agree with the trend observed for capsids, where NCs resulted in well-ordered shells compared to nonuniform NPs.

Beyond surface ligand-mediated internanoparticle interaction, functional groups such as carboxylic acids can be exploited for metal coordination-directed self-assembly of NPs.^91^ Chandra et al. reported metal coordination-induced self-assembly of glutathione (GSH) capped AuNCs.^92^ By controlling the concentration of divalent metal ions (Cs^+^, Mn^2+^, Pb^2+^, Cd^2+^, Sn^2+^, Zn^2+^, Fe^3+^, Al^3+^, and Sn^4+^), the size of the spherical superstructure was tuned from 30 to 200 nm (Figure 5d,e). Among all tested divalent metal ions, Sn^2+^ showed more stable self-assembled structures. The resulting spherical particles significantly increased the photoluminescence quantum yield (PLQY), photocatalytic efficiency, and biological properties. For example, the PLQY was increased from 3.5% in individual NCs to 25% in self-assembled structures (Figure 5g,h). Furthermore, the photocatalytic efficiency using a model dye degradation experiment showed that in UV irradiation at 350 nm wavelength the degradation of methylene blue occurs within 5.5 min in the presence of self-assembled structures. However, the degradation occurred at 112 min for AuNCs and 140 min when no catalyst was used. Furthermore, the superstructures displayed better bioavailability than individual AuNCs. Better insights into the structure were needed to understand the amplified PLQY, catalysis, and bioavailability. Cryo-TEM imaging and electron tomographic reconstruction revealed the spherical nature of the superstructures. The cross-sectional view of 3D reconstruction revealed a densely packed network of AuNCs, resulting in a framework-like structure with a regular order (Figure 5f). The metal–ligand (Sn^2+^–GSH) interactions induce a well-defined network prohibiting several nonradiative relaxation modes in the frameworks. The strong luminescence primarily arises from the highly luminescent T1 state to Au (0) HOMO with an enhanced ligand-to-metal-to-metal charge transfer (LMMCT) relaxation mechanism.

The reversible self-assembly of NCs using noncovalent interactions is well-documented in the literature.^93^ However, dynamic covalent bonding has not been explored for reversible NP self-assemblies. Lakshmi et al. reported the dynamic covalent chemistry driven by [2 + 2] photocycloaddition-mediated reversible self-assembly of Au_25_ NCs.^94^ When thiolated umbelliferone (7-hydroxycoumarin) ligands capped AuNCs irradiated with UV light at 365 nm, the coumarin ligands of neighboring nanoclusters undergo [2 + 2] cycloaddition reaction facilitating inter-NC bonding via covalently linked cyclobutene adducts (Figure 5i). TEM, STEM, and 3D reconstruction suggested toroid formation (Figure 5j-m). Using tomography reconstruction of the early stages of assemblies, it was shown that initially, the AuNCs form spherical framework assemblies. Continued irradiation led to the fusion of a spherical structure and elongation, resulting in toroids. The toroidal outer diameter varied from 500 nm to 3.0 μm, and the rim thickness up to 140 nm. TEM tomography of the toroid shows that the rim is composed of densely packed NCs. More importantly, further irradiation led to the fusion of toroids into honeycomb-like supertoroidal macroscopic frameworks. Due to the dynamic nature of the [2 + 2] cycloaddition reaction, irradiation of toroids at 254 nm resulted in disassembly into individual NCs. The reversible nature of the cycloaddition reaction was exploited for the conjugation of 5-fluorouracil and photocontrolled release.

**Figure 6:**
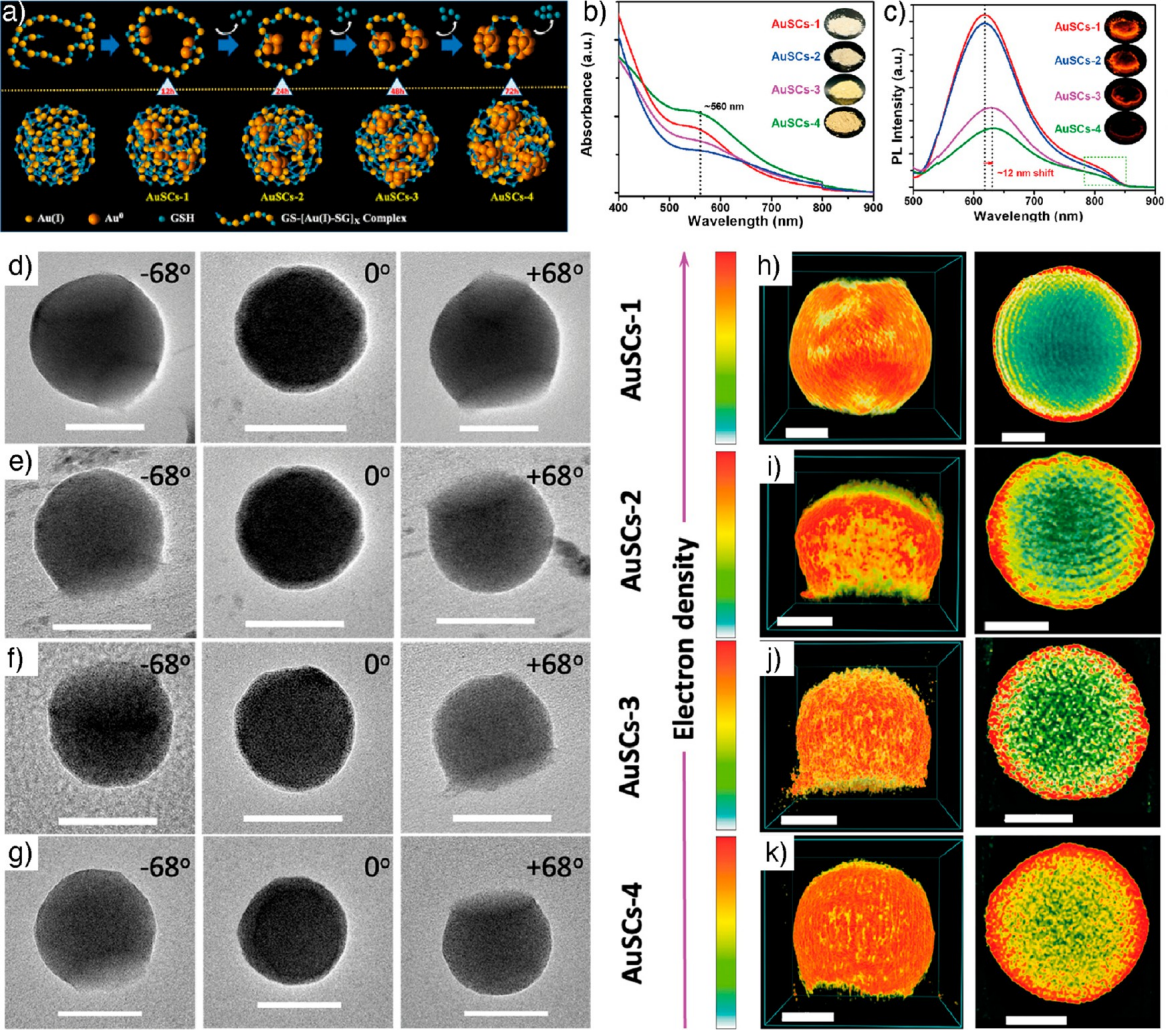
*In situ* depletion guided assembly of gold superclusters. (a) Schematic representation of the *in situ* depletion guided nanoshell-like AuSC formation. (b) Absorbance spectra of AuSCs. (c) PL intensity of AuSCs. (d–g) TEM images of AuSCs at different tilt angles indicate spherical morphologies. (h–k) 3D reconstructed structures of AuSCs (left) and their cross-sectional views (right) showing differences in core–shell structures of AuSCs. Reproduced with permission from ref (95). Copyright 2023 American Chemical Society.

Despite the atomic-level precision of NCs, their self-assembly often leads to nonuniform superstructures or heterogeneous self-assembled end products. Like individual building blocks, control over the size and shape of the superstructures influences their optical, biological, and catalytic properties. Therefore, improved methods to prepare highly uniform NC superstructures are needed. In this context, Bera et al. developed nanoshell-like assemblies called “superclusters” (SCs) using *in situ* depletion-guided engineering of GSH-capped AuNCs (Figure 6a).^95^ The Au(I) thiolate complexes were mixed with a high percentage of polyethylene glycol (PEG-600) as a depletant, which resulted in spherical assemblies of an average diameter of 110 ± 10 nm. By maintaining constant depletion, the formation of metallic Au core was triggered by sacrificing the GSH ligands from the Au(I)-thiolate complexes using thermal activation of superstructures. The NC density was tuned by controlling the thermal treatment times at 12, 24, 48, and 72 h. Accordingly, the optical properties of the AuSCs were tuned. For example, at 12, 24, and 48 h, treatment resulted in AuSCs-1, AuSCs-2, and AuSCs-3, respectively, displaying typical absorbance spectral features of AuNCs (Figure 6b).

However, AuSCs-4, treated for 72 h, showed an interesting surface plasmon resonance peak (Figure 6c). Furthermore, the PL intensity also showed that AuSCs-1 and AuSCs-2 displayed PL intensities higher than those of AuSCs-3 and AuSCs-4. The question arises whether the surface plasmon peak is due to AuNP formation or strong NC-NC interaction under a confined environment. The AuSCs were tested for their peroxidase-like catalytic activity using a colorimetric assay based on 3,3′,5,5′-tetramethylbenzidine (TMB) oxidation. All AuSCs displayed peroxidase-like activity by initiating the reaction within 5–10 min. However, the results suggested AuSCs-4 displayed higher catalytic activity, 33.5-fold higher than AuSCs-1. To gain insights into the origin of the surface plasmon resonance and the difference in catalytic activity, a 3D reconstruction of all AuSCs was performed. All AuSCs displayed spherical morphologies (Figure 6d–g). Interestingly, from cross-sectional views, it was evident that in AuSCs-1, only the surface Au(I)-thiolates converted into AuNCs, with an amorphous interior and a thin AuNC shell (Figure 6h–k). The shell thickness and the density of NCs increased from AuSCs-1 to AuSCs-4. Therefore, it was concluded that the surface plasmon resonance peak arises from the strong interaction of AuNCs in a confined shell and is not due to plasmonic NP formation.

## 4 Nanoparticle–Nanocluster Composites

Hybrid and composite nanomaterials offer possibilities to engineer materials with tunable and controllable functions, properties, and applications. Anisotropic AuNPs such as nanorods (AuNRs) and nanotriangles (AuNTs) display unique surface plasmon resonance peaks, sensitivity to their surrounding chemical environments, and act as optical antennas for conjugated dye molecules.^96,97^ Importantly, fluorescent organic dye-conjugated NPs have been shown to alter the luminescence properties of the dye molecules. Luminescent hybrid materials offer multimodal imaging, sensing, drug delivery, and photodynamic therapeutic applications.^91^ For example, AuNRs with selective tip-functionalization with fluorescent dye molecules have been shown to display a 10-fold increase in luminescence compared to fluorescent dye itself.^98,99^ However, organic dyes undergo degradation and photobleaching. On the other hand, semiconductor quantum dots emerged as luminescent nanomaterials with excellent PLQY.^100^ However, they are toxic and not suitable for bioimaging. Silica quantum dots, on the other hand, are nontoxic but prone to oxidation.^101^ Because of their low toxicity and high photothermal stability, noble metal NCs have emerged as interesting luminescent nanomaterials.^91,92^ Furthermore, combining plasmonic NPs with atomically precise NCs offers unique plasmon-exciton coupling. Therefore, developing methods to fabricate NP-NC composites and hybrids will pave the way for a new type of nanomaterial with enhanced optoelectronic properties.

**Figure 7:**
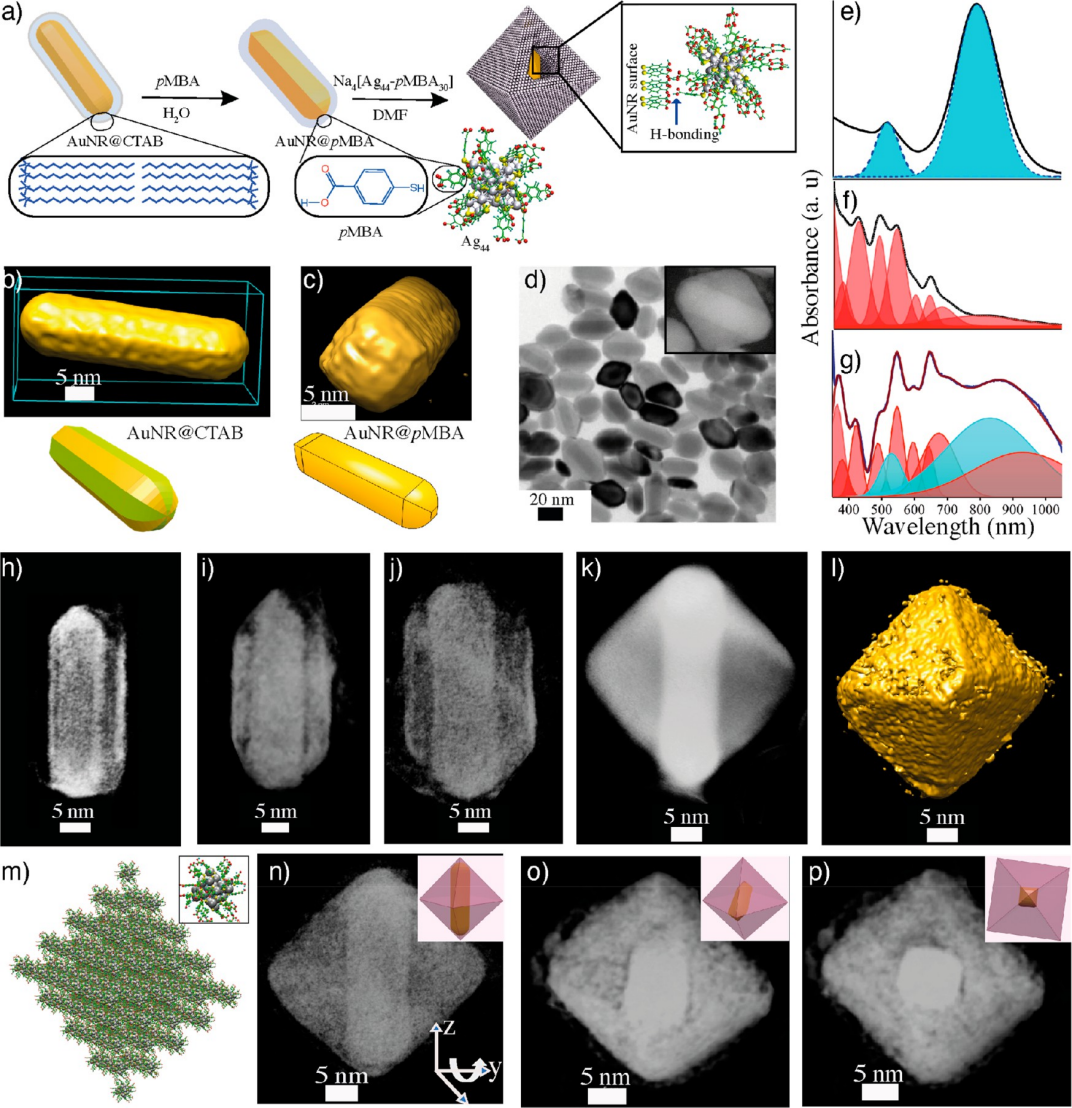
Composite cages. (a) Schematics showing the structure of AuNR@CTAB, AuNR@pMBA, and AuNR-Ag composite cage. (b) 3D reconstructed structure of AuNR@CTAB. (c) 3D reconstructed structure of AuNR@*p*MBA. (d) TEM image of composite cages (inset showing DF-STEM image of an individual cage). (e–g) Absorbance spectra of AuNR, Ag_44_, and composite structures, respectively. (h–j) 3D reconstructed structures of composites showing different intermediate stages of the growth. (k and l) DF-STEM 3D reconstructed structures of composite cage showing octahedral morphology. (m) Computational simulation showing the interaction of Na_4_Ag_44_pMBA_30_. (n–p) 3D reconstructed structure viewed at different orientations, showing the location of AuNR in the composite cage. Reproduced with permission from ref (104). Copyright 2018 John Wiley & Sons.

Som et al. reported the formation of composite bilayered structure when titanium nanowires interacted with atomically precise AgNC, Na_4_Ag_44_-*p*MBA_30._ Importantly, Na_4_Ag_44_-*p*MBA_30_ shows patchy hydrogen bonding bundles.^102^ This property has been utilized to develop macroscopic, mechanically robust, strong, and elastic monolayer membranes.^103^ In its solid-state structure, Na_4_Ag_44_-*p*MBA_30_ displays bundles of two (L2) and three (L3) ligands. The L2 bundles allow intralayer hydrogen bonding and L3 form interlayer hydrogen bonding. Chakraborty et al. investigated the hydrogen bonding directed AuNR-Na_4_Ag_44_-*p*MBA_30_ self-assemblies into composite cages (Figure 7a–d).^104^ The CTAB-protected AuNRs (*d* ≈ 10 nm, *l* ≈ 30 nm) were exchanged with *p*MBA ligands (AuNR@*p*MBA). The self-assembly was achieved by mixing the AuNR@*p*MBA and Na_4_Ag_44_-*p*MBA_30_ in *N*,*N*-dimethylformamide (DMF). The *p*MBA ligands on the surface of AuNR and AgNC allow the NR-NC interaction via hydrogen bonding. It was shown that the resulting AuNR-AgNC composite displays the peaks arising from the AuNR and AgNCs in their UV–vis spectra (Figure 7e–g). This suggests that the intrinsic properties of both components were retained in the composite structures. However, significant broadening was observed in the NIR region of the spectrum, presumably due to possible electronic interactions between AuNR and NCs in the composites. The conventional TEM and STEM images show AuNRs in the core and NCs in the shell of the self-assembled structure. TEM and STEM tomographic reconstruction revealed that AuNR-AgNC coassembly resulted in an octahedral cage (Figure 7h–p). Notably, each cage encapsulated a single AuNR, offering a rapid and robust approach to composite supracolloidal cages.

The pure Na_4_Ag_44_-*p*MBA_30_ crystallizes in a triclinic lattice. The crystal structure data were used for computational simulations to understand the octahedral nature of the composite cage. The simulation results suggest that the lattice structure of octahedral assemblies is face-centered cubic. Tomographic reconstruction of different stages of growth suggests that in the early stages of the assemblies Na_4_Ag_44_*p*MBA_30_ formed a uniform assembly around the entire AuNR surface (Figure 7h). As the reaction proceeds, the AgNCs preferentially interact around the AuNR body compared to the tip or the two ends (Figure 7i,j). This is attributed to a higher density of hydrogen bonding sites at the center than the AuNR tips. Furthermore, the preferential attachment of AgNCs to the Au ⟨110⟩ than Au ⟨100⟩ facets of GNR@pMBA, induces anisotropic growth, resulting in octahedral nanocages encapsulating a single AuNR. The NP–NC interactions can be controlled by modifying the functional groups of the ligands. For example, partially deprotonated *p*MBA-capped AuNCs, Au_102_-*p*MBA_44_ and Au_250_-*p*MBA_n_ NCs interacted with AuNR@*p*MBA in aqueous media. Unlike Na_4_Ag_44_-*p*MBA_30_, the AuNCs produced a monolayer shell around AuNRs. This is due to the negatively charged carboxylates on the NC surface, which provided sufficient electrostatic repulsion to stabilize the composite structures. Therefore, by controlling the ligand functional group and reaction media, the composites’ shell thickness and morphological features can be tuned.

Self-assembly of metal nanoparticles mediated by the noncovalent interactions between the surface ligands allows detailed investigation of the structural, morphological, and compositional effects on hybrid and composite structure formation. Chakraborty et al. investigated the interaction of AgNCs of different ligand functionalities such as hexadecyltrimethylammonium chloride (CTAC), dimethylbenzenethiol (DMBT), 1,2-bis(diphenylphosphino)ethane (DPPE) with CTAC capped AuNTs.^105^

For example, when Ag_25_DMBT_18_ interacted with CTAB-capped AuNTs, the formation of Ag-doped AuNTs was observed with the etching of Au atoms from the tips of triangles. Interestingly, dendritic shells of Ag were formed around AuNTs when they were mixed with Ag_25_H_22_DPPE_8_. The etching of Au atoms was found to be affected by the type of ligand on the AgNC surface. For example, faster etching was observed when AuNTs interacted with Na_4_Ag_44_-*p*MBA_30_. In contrast, the directional hydrogen bonding prevents atom exchange or etching, resulting in a stable composite core–shell structure. This was supported using composite formation using AuNT@*p*MBA and Na_4_Ag_44_-*p*MBA_30_ NCs, which resulted in a core–shell structure without any etching or doping.

**Figure 8:**
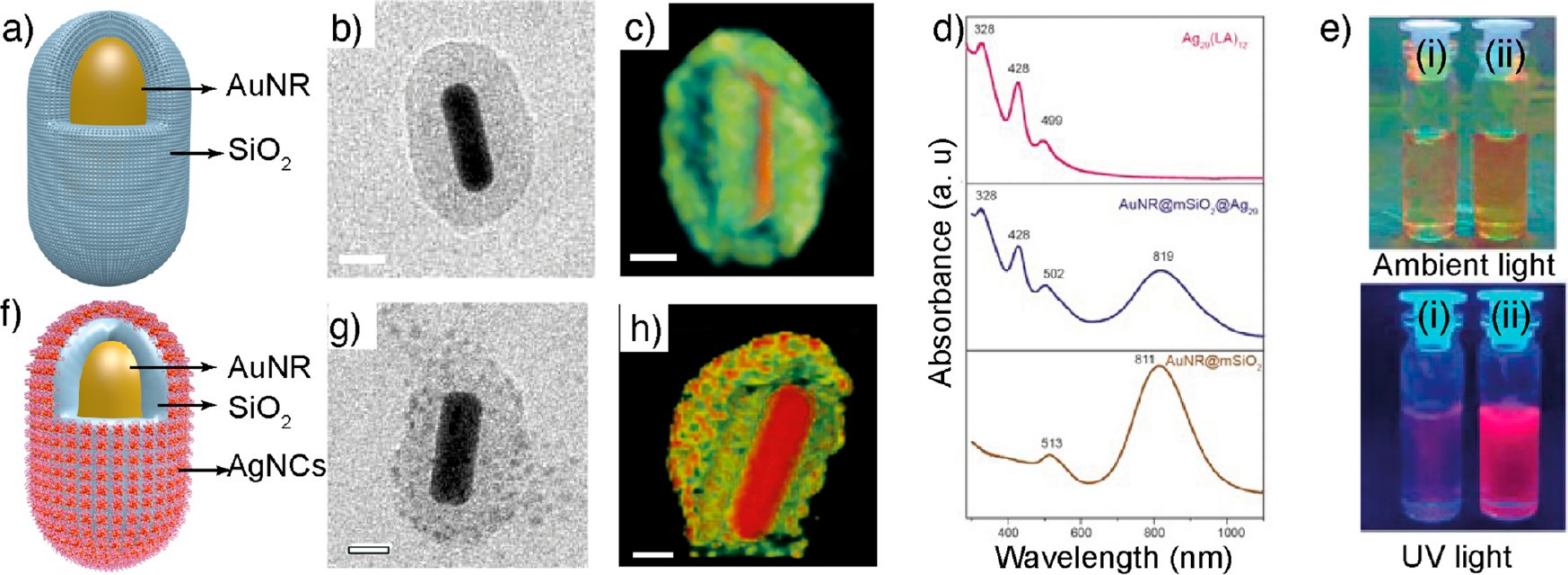
Shell isolated composites. (a) Schematic illustration of a silica coated AuNR (AuNR@SiO_2_). (b) TEM image of silica coated AuNR@SiO_2_. (c) 3D reconstructed structure of AuNR@SiO_2_. (d) Absorbance spectra of Ag_29_LA_12_, AuNR@SiO_2_@Ag_29_, and AuNR@SiO_2_. (e) Photographs showing solutions of AuNR@SiO_2_ (i) and AuNR@SiO_2_@Ag_29_ (ii) under ambient light (top) and UV irradiation (bottom). (f) Schematic illustration of silica coated AuNR@SiO_2_@Ag_29_. (g) TEM image of AuNR@SiO_2_@Ag_29_. (h) 3D reconstructed structure of AuNR@SiO_2_@Ag_29_. Reproduced with permission from ref (106). Copyright 2022 American Chemical Society.

While doping, etching, and composites discussed above are innovative approaches for multifunctional nanomaterials, they do not have luminescent properties. However, they provide clues to control and tune the interaction between plasmonic NPs and NCs. In this context, Chakraborty et al. reported a three-component system consisting of AuNR and lipoic acid (LA) capped AgNCs (Ag_29_LA_12_) to develop luminescent composites (Figure 8).^106^ To anchor the NCs on the AuNR surface and avoid direct interaction, ligand exchange, or doping, the AuNRs were coated with mesoporous silica. The silica-coated AuNRs (AuNR@SiO_2_) were surface functionalized using (3-aminopropyl) triethoxysilane (APTES), which provides a positive surface charge for electrostatic assembly with negatively charged AgNCs. Furthermore, the coating also improves the photothermal stability of AuNRs and prevents photoluminescence quenching. The interaction between the AuNR and Ag_29_LA_12_NCs was tuned by controlling the thickness of the silica layers (Figure 7a–h). The resulting composites displayed a nearly 2-fold increase in photoluminescence compared to Ag_29_LA_12_ alone (Figure 7e). To understand the effect of coating and the location of NCs, 3D reconstruction was performed for as-synthesized AuNR, AuNR@SiO_2,_ and AuNR@SiO_2_@Ag_29_. The 3D reconstructed structures of AuNR@mSiO_2_ revealed that the silica shell was not uniformly distributed on the AuNR surface. Instead, the AuNR surface facets were protected alternately in all AuNR@SiO_2_ layers irrespective of the silica layer thickness. This is attributed to the difference in the surface energy of different sets of planes of AuNRs. This finding suggests that choosing other particle morphologies with facets such as AuNTs may offer composites with distinct photophysical properties. The 3D reconstruction of AuNR@SiO_2_@Ag_29_ showed that the NCs are anchored on the silica surface and no diffusion was observed.

## 5 Biohybrid Superlattices

**Figure 9:**
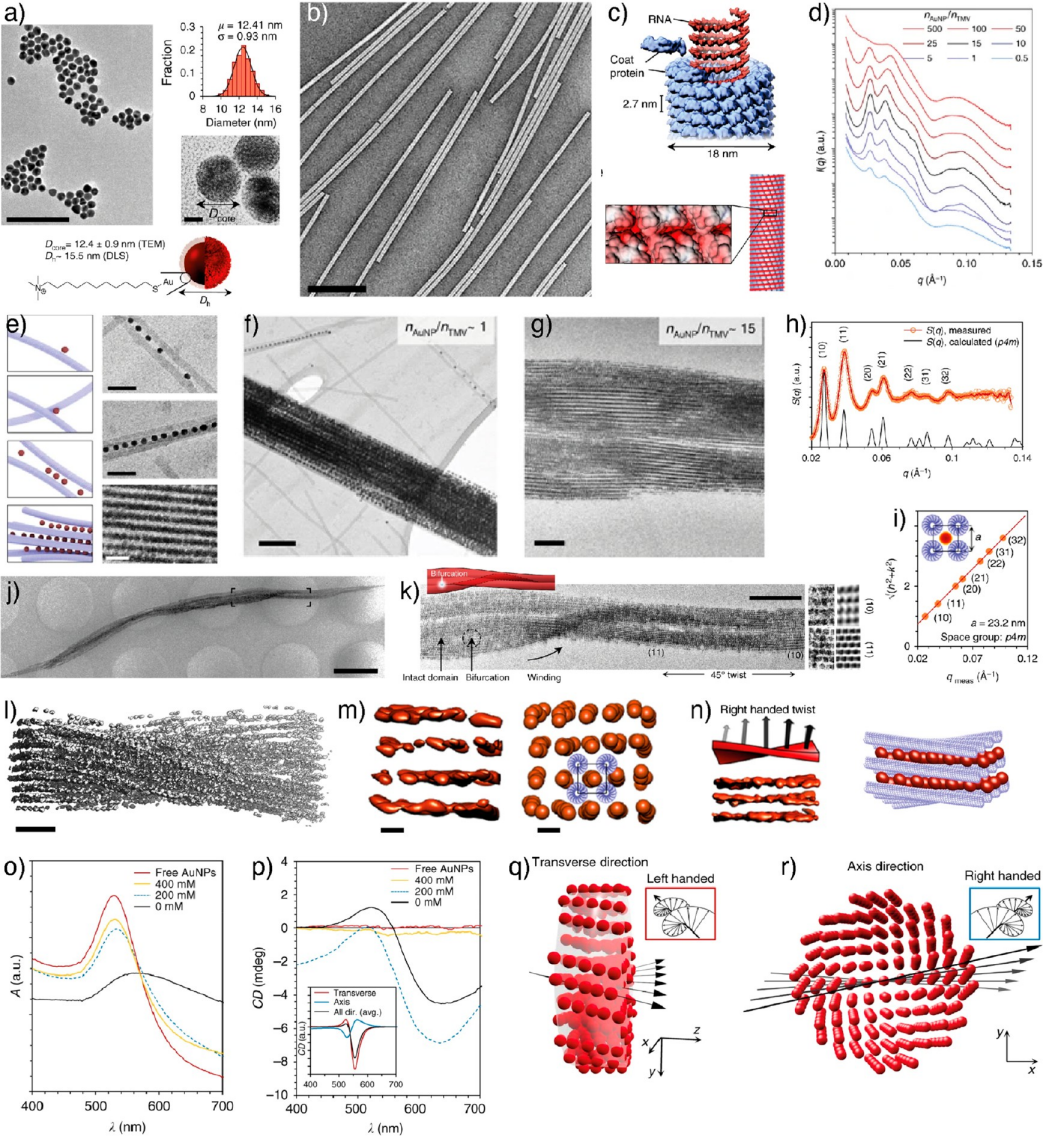
Virus-AuNP superlattices. (a) TEM image, size distribution, and schematic structure of cationic AuNPs. (b) TEM image of negatively stained TMVs. (c) Schematics showing the structure and repeat units of TMV. (d) SAXS patterns were recorded at varying AuNP/TMV ratios. (e) Shows schematics and concentration-dependent cryo-TEM images of the TMV-AuNP cooperative assembly. (f) Cryo-TEM image of a superlattice at *n*_AuNP_/*n*_TMV_ = 1. (g) Cryo-TEM image at *n*_AuNP_/*n*_TMV_ = 15. (h,i) SAXS pattern at *n*_AuNP_/*n*_TMV_ = 15 indicating square lattice. (j) Cryo-TEM image showing right-handed helical twist. (k) Cryo-TEM image used for 3D reconstruction. (l) 3D reconstructed structure of superlattice showing right-handed helical twist. (m) Isosurface view of individual nanoparticle chains and the space occupied by TMVs (indicated in blue). (n) Right-handed twist. (o) Absorbance spectra of AuNPs and AuNP-TMV complexes. (p) CD spectra of AuNPs and AuNP-TMV complexes (inset shows the computationally simulated CD spectra). (q,r) Computational simulations in the transverse direction (left) and the axis direction (right). Reprinted with permission under a Creative Commons license (CC-BY 4.0) from ref (107). Copyright 2017 Nature Publishing Group.

Biological colloidal particles, such as virus capsids, protein cages, and synthetic DNA origamis, are excellent building blocks for hybrid structures.^51^ Their atomically precise structure, well-defined surface functional groups, and patchy interacting sites offer precise control over the structure, morphology, and functionalities. They are excellent templates for long-range-ordered structures and hierarchically complex assemblies across length scales. Liljeström et al. reported a virus particle-AuNP superlattice using controlled electrostatic assembly in aqueous media (Figure 9).^107^ In this study, (11-mercapto undecyl)-*N*,*N*,*N*-trimethylammonium bromide (MUTAB) capped spherical cationic AuNPs of 12.4 ± 9 nm and tobacco mosaic virus (TMV) particles were used (Figure 9a–c). Electrostatic assembly generally leads to uncontrolled aggregation. Therefore, the cationic NPs were first treated with electrolytes, such as NaCl. This resulted in the aggregation of cationic AuNPs. The aggregated AuNPs were treated with intrinsically negatively charged TMVs and were dialyzed against water. AuNP aggregates are disassembled into individual AuNPs upon dilution, facilitating a controlled electrostatic assembly and a stable complex with virus particles. SAXS studies showed clear diffraction peaks across all ratios of *n*_AuNP_/*n*_TMV_ from 0.5 to 500 (Figure 9d). However, the best-resolved peaks were obtained for n_AuNP_/n_TMV_ between 10 and 25. Cryo-TEM imaging suggests that similar structures were formed, irrespective of different n_AuNP_/n_TMV_ ratios. However, in the presence of low AuNPs, free TMVs were observed along with the complexes. Variable concentration cryo-TEM imaging suggests that the superlattices nucleate when TMVs are cross-linked by AuNPs (Figure 9e–g).

The cross-linked complexes attract AuNPs to a higher degree than free TMVs. This allows for the alignment of TMVs and the creation of an interstitial channel that is energetically favorable for AuNPs. According to SAXS data, the hybrid structure showed a 2D array in the superlattice. Rather surprisingly, the structure factor S(q) equaled that of a 2D square lattice with a lattice constant of 23.15 nm, suggesting close packing of the building blocks (Figure 9h,i). This was in contrast to typically observed hexagonal packing patterns for rod-like particles. Therefore, the exact reason and arrangement cannot be understood by using SAXS data. Furthermore, as the ionic strength decreases, the internanoparticle distance also decreases. Interestingly, CD spectra at the visible wavelengths of the superlattices showed a helical plasmonic nature. No CD spectra were observed at high ionic strength, i.e., when the components were not assembled into a superlattice. Further evidence to support the origin of the CD spectra from the superlattice was provided by mechanically shaking the mixture, which showed no CD signal. However, understanding the self-assembly mechanism, the origin of CD spectra, and the 2D square lattice formation required extensive structural investigation. Cryo-TEM imaging and 3D reconstruction of a single microwire revealed a right-handed helical twist with a well-defined pitch length and twist ω (360°/helical pitch) of ∼0.13°/nm (Figure 9j,k). Furthermore, careful analysis of the 3D reconstructed structure showed a 2D square lattice. From the cryo-TEM images and cryo-ET, the lattice constants were determined to be 25 nm (≈ lattice constant *a*) for (10) and 17 nm (≈ *a*/√2) for the (11) lattice planes. These values agreed with the SAXS-based lattice constants of 23.2 and 16.4 nm. The inter-NP distance remained constant for a given n_AuNP_/n_TMV_ ratio and was found to be between 15 and 30 nm. The electrostatic repulsion between AuNPs and attraction between AuNP and TMV control the superlattice formation and inter-NP distance. The weak electrostatic interactions limit the formation of 3D lattices. Furthermore, helical twisting in a 3D superlattice is forbidden, as it breaks the translational symmetry in the direction of the rotational axis.

To further understand the mechanism of helical growth, the 3D coordinates from the cryo-ET reconstruction were collected and used for coupled dipole approximation simulations. For computational simulation 400 AuNPs were arranged in a helical superlattice structure maintaining a lattice constant of 23.15 nm and an interparticle distance of 16 ± 1.6 nm. The simulation reproduced the main features of the experimental CD spectrum (Figure 9o–r). However, there were some mismatch in the width and position of the peak-dip feature between the experimental and simulated CD spectra. The observed mismatch is attributed to the variation in the width and ω of the actual superlattice samples as supported using TEM imaging. Furthermore, the simulations revealed that the CD spectra is dependent on the orientation of the structure. Depending on the viewing direction both right-handed (axis direction) and left-handed (transverse direction) twists can be observed for this type of superlattices.

Finally, the effects of the size and shape of the nanoparticle was studied. Larger nanoparticles bind to four TMV molecules. On the other hand, in smaller NPs, each NP binds to three TMV leading to a hexagonal lattice. Chakraborty et al. extended this approach to demonstrate near-infrared chiral plasmonic microwires using TMV-AuNR superlattice formation.^108^ However, the structural variety of the assemblies achieved using protein cages and capsids is limited due to the selected protein’s predetermined shape, charge, and size. Therefore, exploring whether programmable and modular DNA nanostructures could be equally used to organize AuNPs into well-ordered structures is desirable.

**Figure 10:**
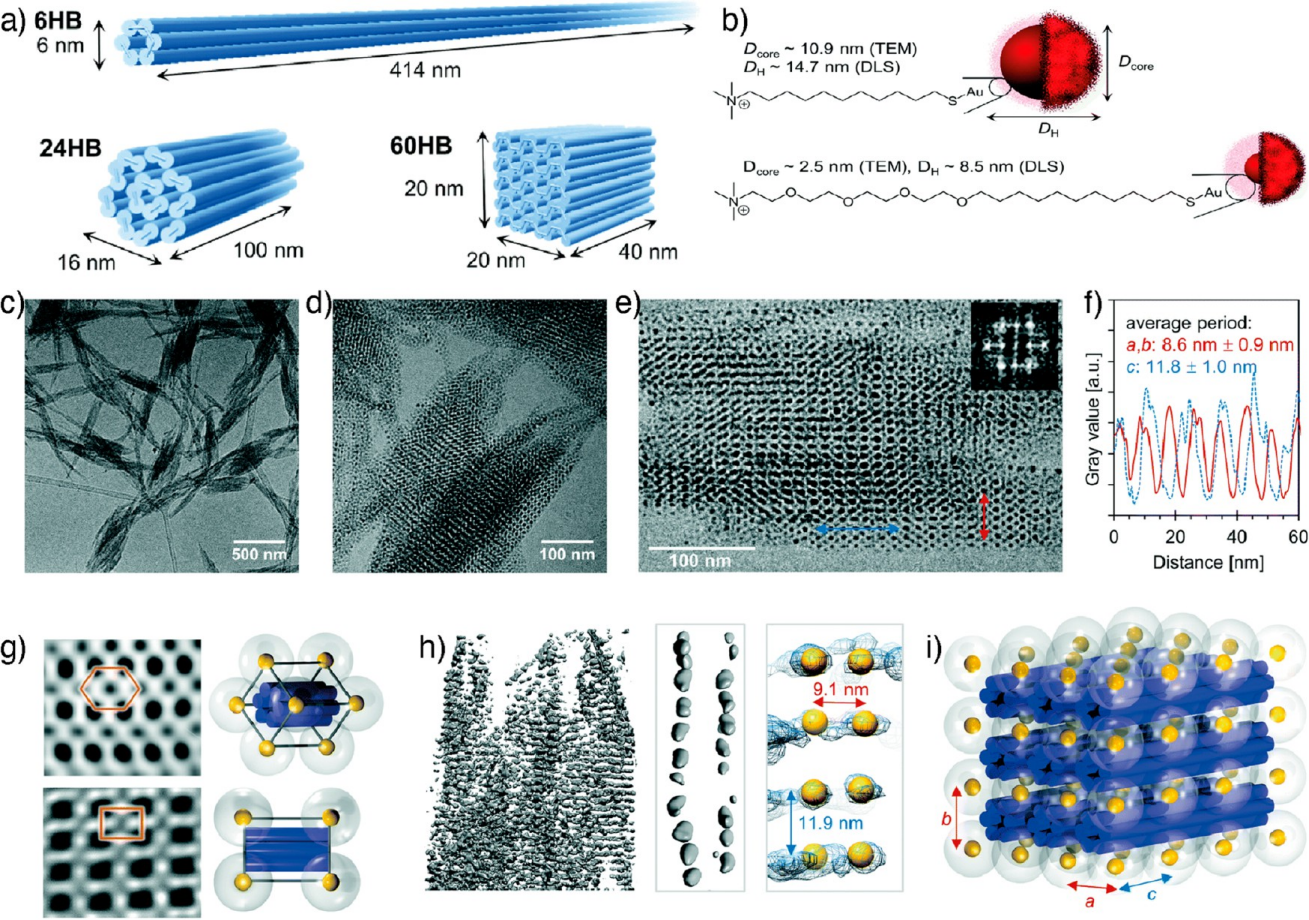
DNA origami-based superlattices and lamellar assemblies. (a) Schematic illustrations of DNA origami structures. (b) Schematic illustrations of cationic AuNPs. (c,d) Cryo-TEM images of 6HB-cationic AuNP superlattices. (e) High-resolution cryo-TEM image of a superlattice (inset shows the FFT). (f) Interparticle distances based on the cryo-TEM image in e. (g) Inverse fast Fourier transform (IFFT) from the cryo-TEM image along different project axes and a schematic of the unit cell. (h) 3D reconstructed structure of superlattice (left), density map showing the arrangement of AuNPs along a single DNA origami (middle and right), and packing patterns of AuNPs along a DNA origami (right) denoted by yellow spheres. (i) Schematic illustration of the 3 × 3 tetragonal unit cell based on 6HB-small AuNP superlattice. Reprinted with permission under a Creative Commons license (CC-BY-NC 3.0) from ref (109). Copyright 2019 Royal Society of Chemistry.

Julin et al. investigated the effect of AuNP size and the shape of DNA origamis in superlattice formation (Figure 10).^109^ Three types of DNA origami structures, viz, 6-helix bundles (6HB), 24-helix bundles (24HB), and 60-helix bundles (60HB), with lateral diameters of 6.0, 16.0, and 28.3 nm, respectively, were used (Figure 10a). Cationic AuNPs of three sizes were used, small, large, and extra large, with diameters of 8.5, 14.7, and 15.8 nm, respectively (Figure 10b). The controlled electrostatic assembly between cationic AuNPs and negatively charged origamis resulted in well-ordered 3D tetragonal superlattices (Figure 10c–i). The small-angle X-ray scattering (SAXS) measurements of aqueous samples containing different combinations of DNA origami and AuNP, as well as varying stoichiometric ratios, *n*_AuNP_/*n*_origami_, revealed well-ordered superlattice structures in the case of 6HB and small AuNP (*d*_core_ = 2.5 nm). Whereas all other studied combinations produced less ordered aggregates with only a short-range order. The cryo-TEM images and 3D reconstruction revealed that 6HB and small AuNPs (*d*_core_ = 2.5 nm) form large, micrometer-sized 3D tetragonal superlattices (Figure 10g–i). The average lattice constants determined from the cryo-TEM images and cryo-ET reconstruction are *a* = 8.6 ± 0.9 nm (sd), *c* = 11.8 ± 1.0 nm (sd) and *a* = 9.1 nm, *c* = 11.9 nm, respectively (Figure 10f). The results were matched with the lattice constants obtained from the SAXS analysis. Surprisingly, superlattices were not formed when these same AuNPs were complexed with either 24HB or 60HB. Larger AuNPs (*d*_core_ = 10.9 nm) could immobilize all three types of DNA origami at *n*_AuNP_/*n*_origami_ ∼ 30–40, indicating an efficient binding between large AuNPs and all studied DNA origami structures. The 6HBs are anisotropic rod-like, flexible particles similar to TMVs. On the other hand, 60HBs do not have a sufficient degree of anisotropy due to their box-like shape limiting dimensional anisotropy and superlattice formation. Therefore, the results suggest that size, shape, and charge complementarity between the building blocks are crucial parameters for superlattice.

**Figure 11:**
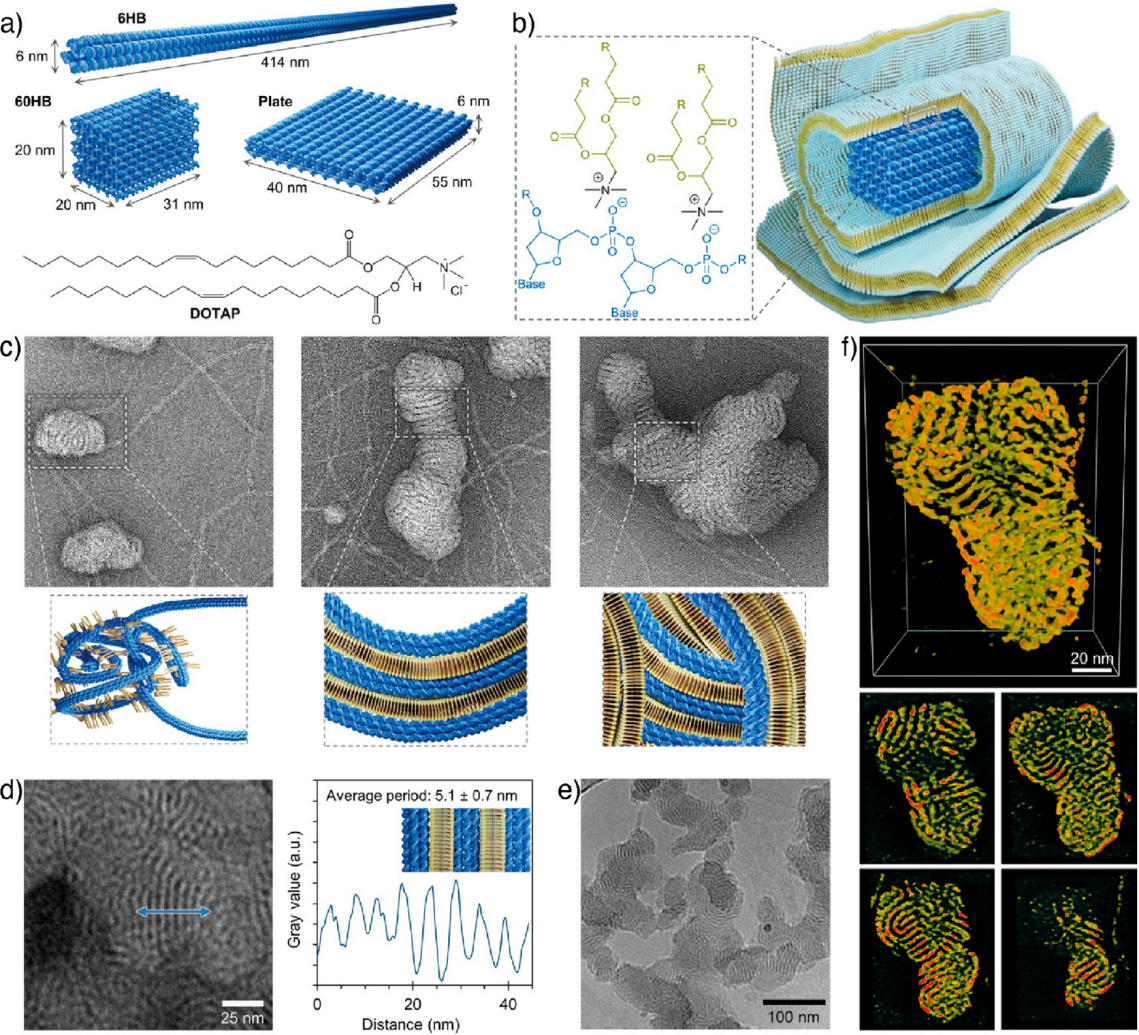
DNA origami-cationic lipid multilamellar structures. (a) Schematic illustration of DNA origami structures and the chemical structure of the DOTAP molecule. (b) Schematic illustration of the multilamellar structure and chemical interaction between negatively charged phosphate groups and cationic lipid molecules. (c) TEM images showing various morphologies of negatively staining 6HB-DOTAP. (d) High-resolution image (left) and interlamellar distance (right). (e) Cryo-TEM image of 6HB-DOTAP complexes. (f) 3D reconstructed structure (top) and cross-sectional views (bottom) of multilamellar structure at different depths. Reproduced with permission from ref (110). Copyright 2021 John Wiley & Sons.

In a recent study, Julin et al. demonstrated the multilamellar structure using electrostatically assembly of DNA origami with a cationic 1,2-dioleoly-3-trimethylammonium-propane (DOTAP) lipid molecules (Figure 11).^110^ Three types of DNA origamis, viz., 6HB, 60HB, and plate-like particles, were used (Figure 11a). The cryo-TEM image analysis showed an average interlamellar spacing of 5.1 ± 0.7 nm, irrespective of the type of DNA origami used. However, tilt series for tomography reconstruction of the vitrified specimens were prone to electron beam radiation damage, which limited the analysis. Therefore, 3D reconstruction was performed using negatively stained specimens (Figure 11c–e).

The 3D electron density map from the TEM tomography reconstruction of the assemblies and their cross-sectional views suggest that the resulting complexes comprise a densely interconnected network (Figure 11f). Interestingly, the 3D reconstruction revelated that concentric lamellar structures were formed when 6HB was used. On the other hand, in the case of 60HB and plate stacked lamellar arrangements were observed. DOTAP forms flat lamellar structure due to zero spontaneous curvature (lipid packing parameter, *P* ≈ 0). However, the observed difference in the lamellar arrangement of hybrid DNA origami-DOTAP structures is attributed to the DNA origami templated lipid packing behavior. For example, the 6HB is a rod-like flexible particle and wraps into a “ball of yarn”-like assemblies with high curvature when combined with DOTAP. On the other hand, 60HB and the plate display low curvature due to their rigid hexahedra structures. The rigid nature of the origami structures promote the stacked lamellar arrangement of DOTAP molecules.

## 6 Biomolecular Assemblies

**Figure 12:**
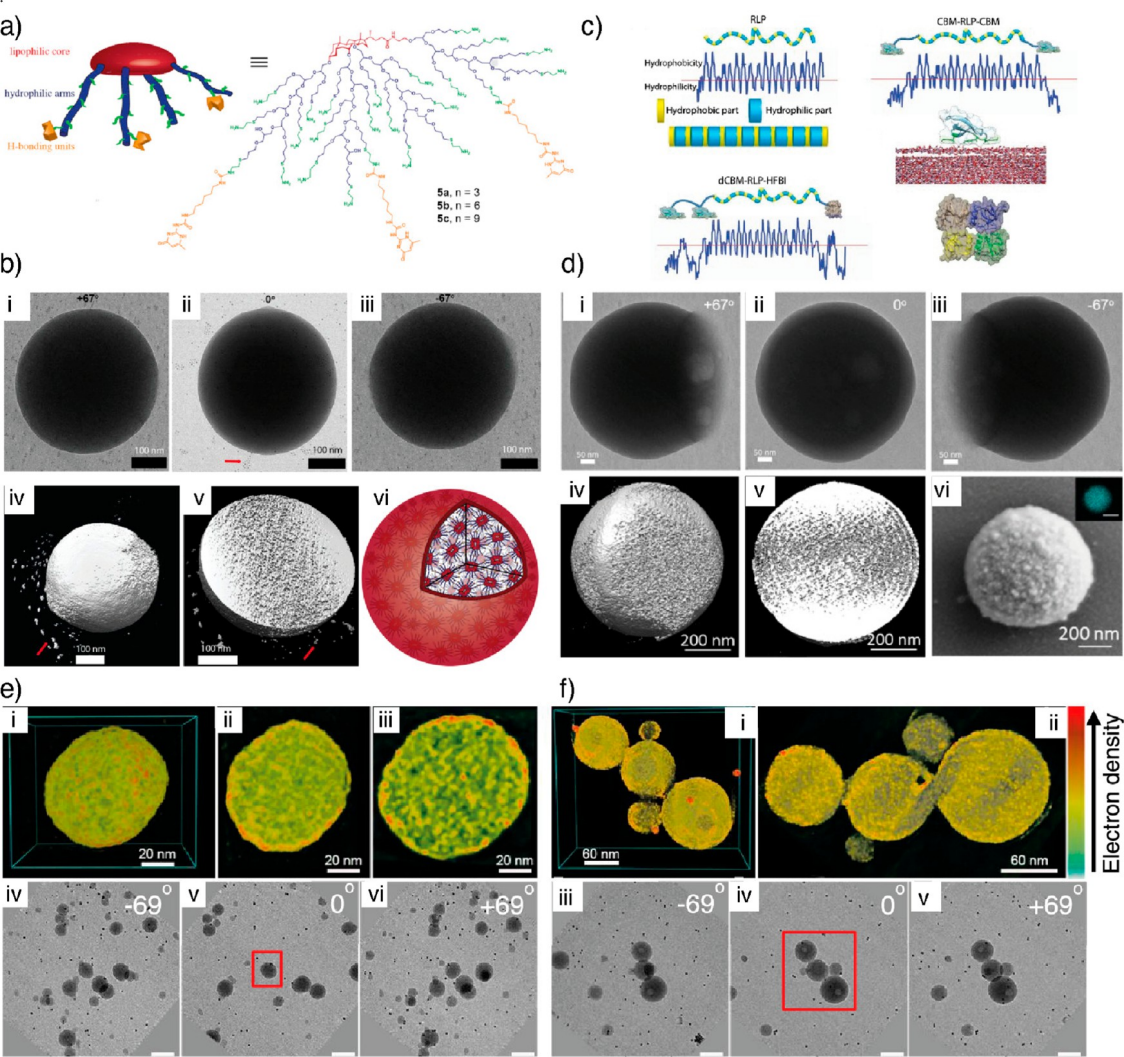
Biomolecular assemblies. (a) Schematic illustration of bile acid-derived star-like amphiphiles. (b) TEM images (i–iii) of a supermicelle at different tilt angles and 3D reconstructed structure, its cross-sectional view, and schematics (iv–vi). Panels (a) and (b) reproduced with permission from ref (112). Copyright 2017 Elsevier. (c) Schematic illustration of CBM appended resilin-like peptides. (d) TEM images (i–iii) of a coacervate at different tilt angles showing spherical morphology and (iv–vi) 3D reconstructed structure, cross-sectional view, and SEM image of a coacervate. Panels c and d reproduced with permission from ref (114). Copyright 2018 Elsevier. (e) 3D reconstructed structures of lignin particles in acetone, cross-sectional views, and TEM images at different tilt angles. (f) 3D reconstructed structures of lignin particles in THF, cross-sectional views, and TEM images at different tilt angles. Panels (e) and (f) reprinted with permission under a Creative Commons license (CC-BY 4.0) from ref (115). Copyright 2021 American Chemical Society.

Unlike metal-nanoparticle-based superstructures, polymer and biomolecular assemblies face several challenges, including specimen preparation artifacts and electron beam-induced damages. Furthermore, the achievable vitrified ice thickness (70–130 nm) also limits cryo-TEM imaging of soft polymeric and biological structures above 100 nm thickness.^111^ Such structures will readily deform, seriously limiting any realistic structural insights. Therefore, alternative specimen preparation methods to preserve the original structures are needed. Bertula et al. studied the self-assembly of star-like amphiphilic derivatives of bile acids conjugated with hydrogen-bonding 2-ureido-4[1*H*] pyrimidinone (UPy) moieties (Figure 12a,b).^112^ The UPy molecules display strong quadrupolar hydrogen bonding.

The star-like amphiphiles self-assemble into nanometric micellar structures in polar solvents such as dimethyl sulfoxide (DMSO). UPy moieties do not form hydrogen bonding dimerization in DMSO, and the self-assembly is due to the intrinsic aggregation behavior of bile acids.^113^ Sequential solvent exchange from DMSO to water via controlled dialysis triggered the hydrogen bonding between UPy units of micelles, resulting in micrometer-sized spherical particles. However, drying artifacts were observed when the specimen preparation was performed under conventional methods. Moreover, they also were not beam tolerant. An alternative approach was then utilized for TEM specimen preparation using the sequential solvent exchange method. In this approach, after placing the sample on a TEM grid, it was sequentially washed with varying ratios of water/methanol, methanol/*tert*-butanol, and finally with *tert*-butanol, followed by vacuum drying. This approach retained the structure and provided specimen stable under electron beam irradiation (Figure 12b,i–v). The tomographic reconstruction showed the spherical nature of the superstructure. Systematic investigation and cross-sectional view suggest the highly interconnected network, i.e., supermicellar structures. A cross-sectional SEM image of spherical particles further supported the dense network, supporting the proposed self-assembly mechanism (Figure 12b,vi).

Fang et al. studied the coacervation of resilin-like peptide fusion proteins containing cellulose-binding terminal domains (Figure 12c).^114^ However, due to their liquid-like nature and the relatively larger size of the coacervates, they tend to deform during cryo-vitrification. This resulted in a flattened structure with cryo-TEM imaging. The cryo-ET reconstruction showed deformed disclike structures with limited structural details. The solvent exchange approach was utilized to understand the morphology of the superstructures. The particles retained their original structure and shape in this process, indicating a spherical nature. Most importantly, the 3D reconstruction of the coacervates revealed layered onion-like structures, with each layer having a lateral width of 20 nm. Each layer was composed of protein subunits. This study provided the first 3D structural details and possible self-assembly mechanistic details of coacervates.

In nature, lignin is another abundant molecule removed as an unwanted waste during pulping and biofuel production. Lignin is a polyphenolic biomolecule with complex and varying chemical structure and molecular weight, making complete structural understanding at the molecular level challenging. However, in recent years, there has been considerable effort to prepare spherical lignin nanoparticles as a sustainable alternative to synthetic polymeric nanoparticles. However, understanding the spherical nanoparticle formation and determination of their 3D structure remain major challenge. Furthermore, it has been shown that the particle morphology depends on the purity and combination of solvents. However, the exact 3D structures and packing are not known. Zou et al. reported an extended study on LNPs prepared from aqueous acetone and aqueous THF (Figure 12 e,f).^115^ The conventional and cryo-TEM images suggested the average sizes of LNPs of 47 ± 13 and 66 ± 22 nm, respectively, in acetone (LNP_acetone_) and THF(LNP_THF_). The electron tomography reconstruction of LNP_sacetone_ and LNPs_THF_ confirmed its spherical nature. The cross-sectional views of the tomographs revealed that the LNPs are composed of homogeneously distributed smaller building blocks (Figure 12e,f). This study provided the first high-resolution 3D structural insight into LNPs. More importantly, it also verified that the LNPs are more compact structures than commonly proposed core–shell structures or hollow structures. Further support on the structure and porosity was provided using SAXS and the nitrogen gas (N_2_) adsorption–desorption method.

## 7 Limitations and Future Perspectives

TEM tomography is a powerful technique for imaging individual nanoparticles, self-assembled structures, and hybrid materials. This Perspective provides insights into some representative examples based on the author’s contribution to understanding the structure, packing patterns, self-assembly mechanism, and crystal structure determination of self-assembled colloidal superstructures. Despite the tremendous progress in instrumentation, imaging, and imaging processing, TEM tomography has several challenges and limitations. Tomography utilizes a series of 2D projections of objects collected by tilting the specimen with an increment in angle or slope. Since multiple images of the objects are to be collected, the sample is exposed to a relatively longer electron beam. This causes radiation damage in soft colloidal particles and biological samples. Low-dose imaging combined with fast tomography methods developed by Bals and co-workers can overcome radiation damage.^76^ In a recent study, Marchetti et al. reported the templated self-assembly of branched Au nanoparticles.^116^ 3D reconstruction of the superstructures using high-angle annular dark-field scanning transmission electron microscopy (HAADF-STEM) imaging and fast tomography revealed the high-resolution internal structure and core–shell nature of the superstructure. Cryo-vitrification (plunge and high-pressure freezing) and automated image collection for aqueous samples also help to minimize radiation damage. However, the cryo-TEM specimen preparation can be laborious. The low-dose imaging, fast image acquisition methods using continuous rotation, and tomography approaches using limited data coupled with neural network reconstruction have been used to overcome the beam damage.^117−119^ However, such methods may encounter a poor signal-to-noise ratio of images and final reconstruction. Direct electron detection cameras (DED) offer imaging with high sensitivity, signal-to-noise ratio, and resolution.^120^ However, the size of data accumulation is relatively large in terabytes. Therefore, data handling, storage, and processing may face challenges. Liquid cell TEM is another emerging technique to study nanoparticle dynamics and self-assembly in their native environment.^121^ However, such experiments require highly specialized liquid cell holders and microelectromechanical system-based (MEMS) chips. They also suffer from background noise, low signal-to-noise ratios, limited tilt range due to narrow visualization windows, and a long image acquisition time. Wang et al. recently showed that combined liquid cell TEM imaging and fast tomography allow high-resolution 3D reconstruction of CTAB-capped AuNRs.^122^ By comparing the 3D reconstruction of AuNRs, it was shown that the internanoparticle distance significantly differs in dry state and liquid state. Introducing this approach to study self-assembly offers more realistic details about the dynamics of such assemblies in real-time in their native environment.

The fundamental limitations of TEM, such as specimen thickness and field of view, may limit the acquisition of high-resolution data from large self-assembled particles. Furthermore, the tilt range, increment angles, and total number of projections directly impact the resolution of the final ET reconstruction. Therefore, the sample should be thin enough to obtain meaningful structural details. For example, the thickness of an object will increase by a factor of , 2, and 3 upon tilting the specimen at angles of 45°, 60°, and 70°, respectively. The increased thickness poses challenges in determining the correct focus to image the object and contributes to artifacts in the final reconstruction. Currently, the method is well-suited for samples up to 30–50 nm thick, but 100 nm is the upper limit. However, large samples require other approaches such as embedding and sectioning using a microtome. For biological samples, high-pressure freezing and cryomicrotome have been used. Some examples discussed in this paper, such as supermicelles and coacervates, can be reconstructed despite their larger size. This has to do with the fact that polymer-based materials are not as dense as metal nanoparticles. Furthermore, they are transparent to electron beams due to their intrinsically porous structure. However, they are not devoid of artifacts that naturally arise from the missing wedges.

Limited tilting angle results in a missing wedge and severely degrades the spatial resolution of ET along the direction of specimen thickness. To overcome the limited angle, dual-axis tilt approaches have been developed. However, dual-axis tilt can improve the resolution only by a factor of .^123^ For larger samples, it is useful to utilize multiple other techniques, including SEM tomography, sectioning, and X-ray microtomography. Despite these limitations and challenges, TEM tomography is one of the most valuable methods for the 3D structure of materials at the nano- to subnanoscale. Over the past few years, there has been tremendous progress in overcoming the above challenges. From continuous fast image collection, direct electron detectors and new algorithms for image reconstruction together with improved computation power resulted in high-resolution reconstruction. While TEM tomography is a revolutionary technique, it is even more powerful when combined with or as a complementary tool to other analytical techniques such as small-angle X-ray scattering. Nanoparticle self-assemblies are excellent model systems to study *in situ* liquid cell-based imaging. Research in this direction has already taken significant steps. Integrating real-time imaging with tomography reconstruction will offer profound insight into the nucleation and growth mechanisms of colloidal self-assemblies under native reaction conditions. Because of their diverse sizes, shapes, and properties, nanoparticles display different dynamics and phase behavior at the interface. In this context, computational simulation methods offer valuable information to understand their properties. Various simulation methods, including Monte Carlo, molecular dynamics, mesoscale simulations, self-consistent mean field theory, and *ab initio* molecular dynamics methods, have been utilized to study the interfacial properties of nanoparticles.^124^ The results from computational simulations can be effectively utilized to validate the experimental results using dry-state, liquid-state, and cryo-TEM-tomography-based 3D structures of nanoparticle assemblies. A combination of multiple image acquisition methods, advanced image processing, and computational methods has the potential to offer high-resolution structural details of self-assembled nanoparticle superstructures. Such methods are also useful for studying soft biopolymer-based assemblies such as coacervates.
